# Retraction: STK35 gene therapy attenuates endothelial dysfunction and improves cardiac function in diabetes

**DOI:** 10.3389/fcvm.2023.1208579

**Published:** 2023-05-09

**Authors:** Frontiers Editorial Office

A Retraction of the Original Research Article STK35 gene therapy attenuates endothelial dysfunction and improves cardiac function in diabetes By Joladarashi D, Zhu Y, Willman M, Nash K, Cimini M, Thandavarayan RA, Youker KA, Song X, Ren D, Li J, Kishore R, Krishnamurthy P and Wang L (2022). Front. Cardiovasc. Med. 8:798091. doi: 10.3389/fcvm.2021.798091

The journal retracts the 13 January 2022 article cited above.

Following publication, concerns were raised regarding the integrity of the images in the published figures. Image duplication concerns were identified in Figures 3D, 4E, 6. The authors failed to provide a satisfactory explanation during the investigation, which was conducted in accordance with Frontiers’ policies. As a result, the data and conclusions of the article have been deemed unreliable and the article has been retracted.

This retraction was approved by the Chief Editors of Frontiers in Cardiovascular Medicine and the Chief Executive Editor of Frontiers. The authors have not responded to correspondence regarding this retraction.

